# Resistance to thyroid hormone associated with a novel mutation of the thyroid β receptor gene in a four-year-old female

**DOI:** 10.1186/1687-9856-2011-3

**Published:** 2011-06-21

**Authors:** Karina T Canadas, Scott A Rivkees, Robert Udelsman, Christopher K Breuer

**Affiliations:** 1 Department of Surgery, Yale Pediatric Thyroid Center, Yale-New Haven Children's Hospital, 2 Park Street, West Pavilion, 2nd Floor, New Haven, CT 06510, USA; 2 Department of Pediatrics, Yale Pediatric Thyroid Center, Yale-New Haven Children's Hospital, 2 Park Street, West Pavilion, 2nd Floor, New Haven, CT 06510, USA

**Keywords:** child, thyroid, thyroid hormone resistance

## Abstract

Resistance to thyroid hormone (RTH) is a rare syndrome of reduced responsiveness of target tissues to thyroid hormone and is caused mutation in the thyroid β receptor gene. We report a novel mutation, E445X, causing RTH in a 4-year old girl. The patient exhibited extreme signs and symptoms of RTH at an early age, and had a large compressive goiter. Following total extracapsular thyroidectomy, upper airway compression was relieved and symptoms of hyperthyroidism improved. This case appears to be the youngest child recorded to have undergone total thyroidectomy for RTH. Post-operative TSH elevations were managed with every-other-day triiodothyronine therapy.

## Introduction

Resistance to thyroid hormone (RTH) is a rare syndrome of reduced responsiveness to thyroid hormone that is caused by an alteration in the thyroid β receptor gene [1,2]. The incidence of RTH is 1:40,000-50,000 live births and inheritance is autosomal dominant [2,3]. De novo mutation occur approximately 17% of cases, and both males and females are equally affected [2]. RTH is associated with elevated circulating levels of triiodothyronine and thyroxine along with increased levels of thyroid stimulating hormone (TSH) [1,2].

In response to TSH secretion thyroid enlargement occurs in RTH [2]. In some situations, thyroid enlargement may result in upper airway compression. Learning disabilities, developmental delay, tachycardia, attention-deficit-hyperactivity disorder, and delayed skeletal maturation are also seen in RTH [1,2]. We report a 4-year old girl with upper airway compression due to RTH caused by a novel mutation in the thyroid β receptor gene. We also detail post-operative management with every-other-day tri-iodothyronine.

## Case Report

A four-year old female was found to have thyroid enlargement by her primary care provider. At four years of age, she was referred to a pediatric endocrinologist with a large goiter causing compressive symptoms, including sleep apnea and occasional inspiratory stridor. The child had profound hyperkenesis and difficulty with concentration. There was no family history of RTH.

At 4 years of age, the child was at the 40^th ^percentiles for height and weight. The blood pressure was 112/61, and the resting heart rate was 120 beats per minute. A large goiter was present. Ultrasonography revealed a diffusely enlarged gland without extension into the thoracic cavity. The estimated gland volume based on ultrasonography was 150 ml.

The total T3 was > 2,000 ng/dL (normal range 80-180 ng/dL); the total T4 was 15.1 ug/dL (normal range 4-12 ug/dL); the free T4 was 1.8 ng/dL (normal 0.7-1.9 ng/dL); the TSH was 60 mU/L (normal range 0.5-6 mU/L). The skeletal age was 3 years at a chronological age of 3 years, 11 months. A magnetic resonance imaging (MRI) study of the brain did not reveal any brain abnormalities nor pituitary enlargement.

Genetic analysis revealed a novel mutation of the thyroid receptor-β gene, E445X. This mutation introduces a stop-codon in exon 10.

The patient was treated with 25 mg atenolol twice a day, which controlled the tachycardia and reduced the hyperkinetic activity. A trial of triiodothyronine (LT3; 25-75 mcg per day) was not successful, as the child developed abdominal pain, nausea, and worsening of her behavioral symptoms. Thus, surgery was performed to alleviate upper airway compression.

In preparation for surgery the patient was treated orally with methimazole (10 mg per day) and supersaturated potassium iodine solution for two weeks (3 drops, t.i.d). Surgery was performed under general endotracheal anesthesia. The thyroid gland was diffusely enlarged and total thyroidectomy was performed preserving the parathyroid glands, recurrent laryngeal nerves and external branches of the superior laryngeal nerves.

**Figure 1:**
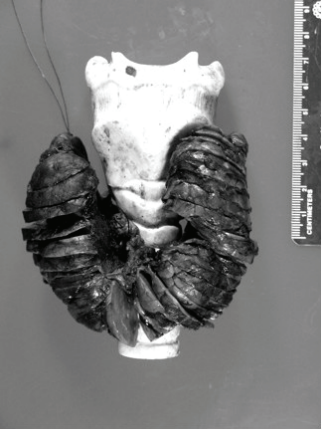
**Gross pathological specimen of the excised thyroid gland**. Specimen was 125 grams. The specimen is place on model of an adult trachea that is 5-times larger than that of a 4-year old.

**Figure 2:**
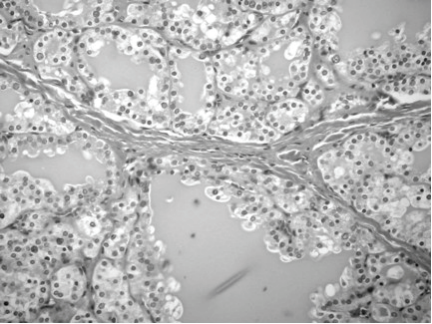
**Histological image showing diffuse hyperplasia**.

Pathological evaluation of the thyroid gland revealed a 125 gm specimen (Figure 1; normal for age 2 gm) [4]. The right lobe measured 8.5 × 5 × 3 cm, the left lobe measured 9.5 × 5 × 3 cm, with an isthmus of 2.5 × 2 × 1.6 cm. Histological examination demonstrated diffuse papillary hyperplasia (Figure 2). Postoperatively, the compressive upper airway symptoms resolved immediately.

Postoperative treatment with LT3 was considered. Due to parental concerns about the previous unfavorable trial of LT3, treatment with levo-thyroxine (LT4) was begun. On large doses of LT4 (175 mcg/day) the T4 was 19.6 ug/dl (252 nmol/L), the free T4 was 210 ng/dl (270 nmol/L); the total T3 was 210 ng/dl (3.2 nmol/L), and the TSH measured 922 uIU/ml.

On the basis of observations in other patients with severe RTH [5,6], LT3 therapy was introduced every other day, with gradual dose escalations every few months. After two years, TSH levels declined from levels ranging between 900 to 1,000 uIU/ml, to about 300 uIU/ml when the every-other day dose reached 200 ug. At this time, the heart rate was about 90 beats per minute. Two year after surgery, a CT scan obtained when the patient suffered mild-head trauma, indicated that pituitary size was normal.

## Discussion

Resistance to thyroid hormone (RTH) occurs as a result from a mutation in the TR β gene that encodes the functionally relevant domain of T3-binding and its adjacent hinge region [1,2]. Our patient exhibited a novel mutation resulting in RTH. E445X is a G to C nucleotide change at position c1339 in exon 10 where the normal glutamic acid at position 445 of the protein is changed to a stop codon. This mutation is expected to result in a truncated TRβ protein molecule.

Goiter development in RTH occurs as the result of increased TSH secretion, and possibly increased TSH action in RTH [1,2]. Medical therapy aims to suppress TSH using daily LT3 therapy [1,2]. However, although the child already had extremely elevated levels of T3, it was not possible for the child to tolerate LT3 therapy. Considering the compressive nature of the large amount of thyroid tissue, surgery was performed.

Thyroidectomy for RTH has been performed in other children whose symptoms and goiter size cannot be controlled medically [7-11]. The youngest individual reported to have thyroidectomy for RTH that we are aware of was a 9 year-old child homo-/hemizygous for TR β gene mutation, with a goiter causing compressive symptoms [11]. That individual exhibited severe psychomotor developmental delay, hearing loss, visual impairment, chronic otitis media and tachycardia. She went onto develop a recurrent goiter, requiring further surgical intervention at ages 13 and 16 [11].

To reduce this risk of goiter recurrence, we performed an extracapsular total thyroidectomy. At two years after surgery, no thyroid tissue was observed in imaging studies.

Because of markedly elevated TSH levels and failure of high-dose LT4 to results in significant TSH level reductions, the child was treated with escalating doses of LT3, based on a reports showing that this strategy helps promote TSH level reductions, while attenuating the adverse effects of LT3 [5,6]. After about two years, when the LT3 dose reached 200 mcg every other day, we observed major decrements in TSH, with no overt symptoms of hyperthyroidism. Although central nervous system imaging studies two years post-surgery did not reveal pituitary enlargement, long-term evaluation will be needed to assess if pituitary hyperplasia occurs. Interestingly, even in the setting of persistently elevated high TSH levels in individuals with RTH, pituitary hyperplasia occurs seldom [12,13].

In sum, we report a novel mutation in the thyroid β receptor gene leading to a compressive goiter. This appears to be the youngest reported patient treated with total extracapsular thyroidectomy for RTH. We also detail post-operative management with every-other-day LT3 with good results.

## Consent

Written informed consent was obtained from the patient for publication of this case report and accompanying images. A copy of the written consent is available for review by the Editor-in-Chief of this journal.

## Competing interests

The authors declare that they have no competing interests.

## Authors' contributions

KC wrote the manuscript and was involved in the surgical care of the patient. SR helped to edit the manuscript and is responsible for the patients ongoing medical endocrinology care. RU was involved in this patients surgical care. CB helped edit this manuscript and is responsible for this patients ongoing surgical care.

All authors have read and approved the final manuscript.
